# Case report of rhabdomyosarcomatous transformation of a primary gastrointestinal stromal tumor (GIST)

**DOI:** 10.1186/s12885-019-6085-3

**Published:** 2019-09-13

**Authors:** Li Li, Marian Khalili, Gregg Johannes, Praneeth Baratam, William F. Morano, Michael Styler, Wilbur B. Bowne, J. Steve Hou

**Affiliations:** 1 Department of Pathology and Laboratory Medicine, Drexel University College of Medicine, 245 N. 15th Street, Philadelphia, PA 19102 USA; 2 Departments of Surgery, Drexel University College of Medicine, 245 N 15th Street, Suite 7150, Philadelphia, PA 19102 USA; 3 Department of Hematology and Oncology, Drexel University College of Medicine, 245 N. 15th Street, Philadelphia, PA 19102 USA

**Keywords:** Gastrointestinal stromal tumor (GIST), Rhabdomyosarcomatous dedifferentiation, KIT, Imatinib (IM), Tyrosine kinase inhibitor (TKI)

## Abstract

**Background:**

Gastrointestinal stromal tumor (GIST) is the most common primary mesenchymal neoplasm of the gastrointestinal tract. Mutations of KIT and platelet-derived growth factor receptor alpha have been well characterized in GISTs. Patients with KIT mutations are generally sensitive to treatment with tyrosine kinase inhibitors. However, some patients with GIST, while initially sensitive to TKIs, gain resistance in later stages of treatment. Heterologous rhabdomyomsarcomatous dedifferentiation of advanced GISTs after long-term imatinib mesylate (IM) therapy has been reported. In these cases, the underlying molecular mechanism of tumor progression and transformation is unclear.

**Case presentation:**

We report one such patient with rhabdomyosarcomatous dedifferentiation of a GIST without metastatic disease after brief 3-month therapy with IM. The tumor was composed of two distinct phenotypes, a CD117 negative region with rhabdomyosarcomatous differentiation directly adjacent to a CD117 positive classic GIST region. Molecular analysis identified the activating KIT exon 11 mutation in both regions, indicating a common origin for both phenotypes. Additionally, the dedifferentiated component contained two synonymous variants in platelet-derived growth factor receptor alpha and KIT. The increased number of synonymous variants in the rhabdomyosarcomatous region may reflect increased genetic instability of this tumor that may have resulted in the loss of CD117 expression in the dedifferentiated component.

**Conclusion:**

This study adds to the growing consensus that rhabdomyosarcomatous GIST progresses from a common GIST primary tumor. The role of IM in this progression is uncertain; however short duration of IM treatment in this study supports the hypothesis that rhabdomyosarcomatous GIST progression is not a consequence of IM therapy. Furthermore, we provide additional information supporting the observation that CD117 negative rhabdomyosarcomatous transformation maintains the activating KIT variant without KIT expression.

## Background

Gastrointestinal stromal tumors (GISTs) are the most common primary mesenchymal neoplasm of the gastrointestinal (GI) tract. There are approximately 5000 new cases of clinically significant GIST in the United States each year [1]. Arising from the interstitial cells of Cajal (“pacemaker cells”), GISTs most commonly occur within the stomach and small intestine. Rare but aggressive GISTs present with numerous intraperitoneal/serosal-based nodules or liver metastasis. Prognostication depends highly on tumor biology and size [1].

Gain-of-function mutations of KIT receptor tyrosine kinase (RTK) or homologous RTK, platelet-derived growth factor receptor alpha (PDGFRA) drive neoplastic cell growth. Approximately 80–85% of GISTs have activating mutations in KIT or PDGFRA. As a result, overexpression of CD117 (KIT protein) is a relatively specific marker for the diagnosis of GIST [2]. Most frequently, KIT mutations are found in exon 11 (66–71%), followed by exon 9 (13%), exon 13, and exon 17. CD117 immunoreactivity has important therapeutic implications as patients harboring KIT mutations (exon 11) respond more effectively to TKI (imatinib mesylate (IM) or sunitinib malate) treatment [3, 4].

Definitive treatment of GIST remains surgical resection. However, in the IM era, medical management is crucial in the neoadjuvant and adjuvant settings. Based on findings from the Z9001 trial, IM was approved by the FDA for intermediate-high risk GISTs as adjuvant therapy, while many have endorsed its use preoperatively for potentially resectable lesions [5, 6]. Furthermore, patients were found to have improved recurrence-free and overall survival when maintained on adjuvant IM for up to three years [7, 8].

However, some patients who are initially responsive to IM, develop resistance with prolonged treatment. Such resistance is frequently associated with tumor recurrence, new tumor growth, or metastasis, with mostly preserved morphologic and immunophenotypic features. Resistance to IM, likely a result of KIT reactivation, is associated with expansion of imatinib-insensitive KIT mutations, re-proliferation of focal nodular tumors, gain of secondary gene mutations, clonal evolution, sub-clonal selection, and metastasis [9–12]. Gene copy number abnormalities may also be found in TKI resistant/dedifferentiated GISTs [13].

Rarely, primary or metastatic GISTs undergo dedifferentiation after initiation of IM. Dedifferentiation has been described in many tumors, and mostly develops de novo independent of treatment. To our knowledge, only seven published cases have shown heterologous rhabdomyosarcomatous dedifferentiation in advanced GISTs after long term IM therapy (14–54 months) [2, 14, 15] (Table 1). Due to the rarity of rhabdomyosarcomatous dedifferentiation of GISTs, the underlying molecular mechanism of tumor progression and transformation is poorly understood. Our current study adds new molecular findings to this rare entity.

**Table 1:** Amino acid changes in Rhabdomyosarcomatous dedifferentiation of GIST as reported in the literature

Morphology^a^ (dedifferentiated)	Mutation	Amino Acid Change	Reference
Rhabdomyosarcomatous^b^	6 nt deletion	KV558–559del and Trp557Phe	our sample
Rhabdomyosarcomatous^b^	6 nt deletion	KV558–559	Jiang 2015 [14]
Rhabdomyosarcomatous	Missense	V559D	Zheng 2013 [15]
Rhabdomyosarcomatous	Missense	V559D	Liegl 2009 case 1 [2]
Rhabdomyosarcomatous	18 nt deletion	556–574 del	Liegl 2009 case 2 [2]
Rhabdomyosarcomatous	Missense	V559D	Liegl 2009 case3 [2]
Rhabdomyosarcomatous	54 nt deletion	556–574 del	Liegl 2009 case 4 [2]
Rhabdomyosarcomatous	none	PDGFRA exon 18 del	Liegl 2009 case 5 [2]

^a^Morphology: the morphologic findings of dedifferentiated component after TKIs treatment in the metastatic sites and non-metastatic sites including our case^b^

## Case presentation

### Clinical history

The patient is a 75-year-old African American man with past medical history of hypertension, gout, and previous DVT. He initially presented to an outside institution with early satiety, increasing abdominal girth, and 20-pound weight loss. A well circumscribed mass, 9.3 cm likely originating from the greater curvature of the stomach, was seen on CT scan. Subsequently, he underwent an endoscopy May 2017, revealing a rounded, submucosal lesion. Biopsy of the lesion revealed spindle to epithelioid proliferative neoplasm with focal necrosis. Tumor cells showed positive immunoreactivity to CD117, CD34 and SMA (focal), but were negative for S100. The proliferative index Ki-67 was approximately 20% and mitotic figures were greater than 5/50 high power fields. An in-frame deletion of 6 nucleotides in exon 11 of the KIT gene was identified by next-generation sequencing (NGS). Diagnosis was consistent with GIST. He was started on imatinib mesylate, 400 mg daily for 3 months. He was then referred to our tertiary medical center for possible bloodless surgical intervention due to his status as a Jehovah’s Witness, declining the use of potential blood products.

**Fig. 1:**
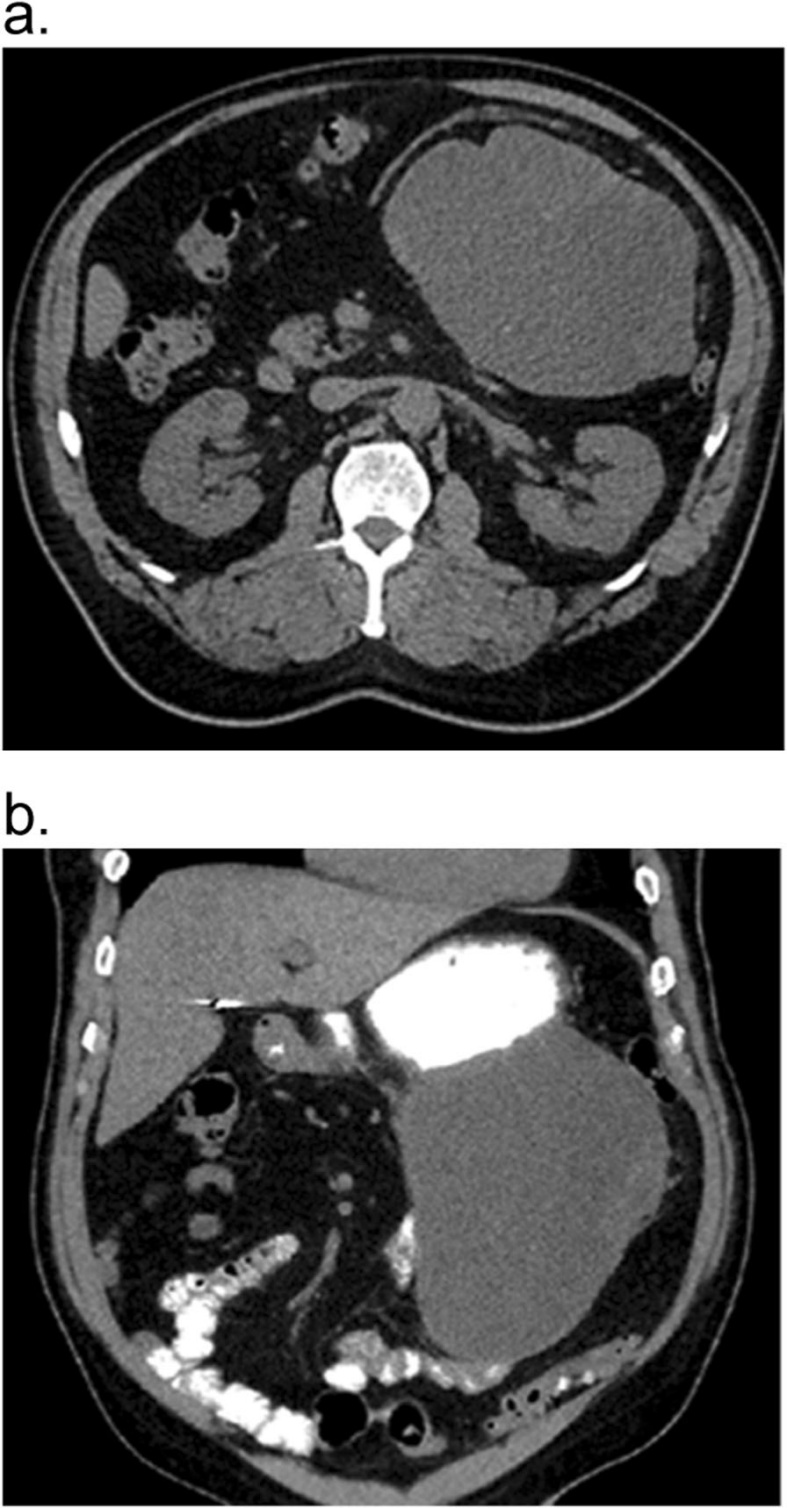
CT-scan of Gastrointestinal Stromal Tumor (GIST) Prior to Surgery: **a** axial, **b** coronal

On repeat imaging (Fig. 1a and b), the tumor was found to have increased in size. The gastric mass appeared adherent to the proximal jejunum, with mass effect on the transverse mesocolon. The decision was made to proceed with surgery in September of 2017. Intra-operatively, a 29 cm friable mass extending from the stomach adherent to the serosa of the proximal jejunum and eroding into the transverse mesocolon was observed. The patient underwent *en bloc* resection including sleeve gastrectomy, proximal small bowel resection, and left hemicolectomy. The patient was subsequently discharged home after resolution of a postoperative ileus. The patient was restarted on Imatinib at a dose of 400 mg daily post-operatively and has since been well tolerated. He has no evidence of disease on repeat CT scan 20 months after the operation.

### Gross and histological diagnosis

The surgical specimen was examined grossly and fixed in 10% neutral formalin. The representative specimen was embedded in paraffin and submitted for permanent histological examination. Four-micron thick hematoxylin and eosin (H&E) stained sections were prepared. Risk stratification was performed according to the recent National Comprehensive Cancer Network (NCCN) guidelines. [16] Immunohistochemical stains for CD117 (rabbit polyclonal A4502, Dako), CD34 (clone QBEnd/10, mouse monoclonal, Ventana), DOG-1 (clone SP31, rabbit monoclonal, Ventana), Desmin (clone DE-R-11, mouse monoclonal, Ventana), smooth muscle actin (SMA) (clone 1A4, mouse monoclonal, Ventana), and MyoD1 (clone EP21, mouse monoclonal, Cell Marque) were performed. Immunostain for myoenin (MYF-4) was performed by Neogenomics.

The resection specimen revealed a 29 × 17 × 5 cm cystic/hemorrhagic lesion arising from the stomach and extending into the retroperitoneum and transverse mesocolon with adherence to the proximal jejunum. No prominent nodular components were seen.

**Fig. 2:**
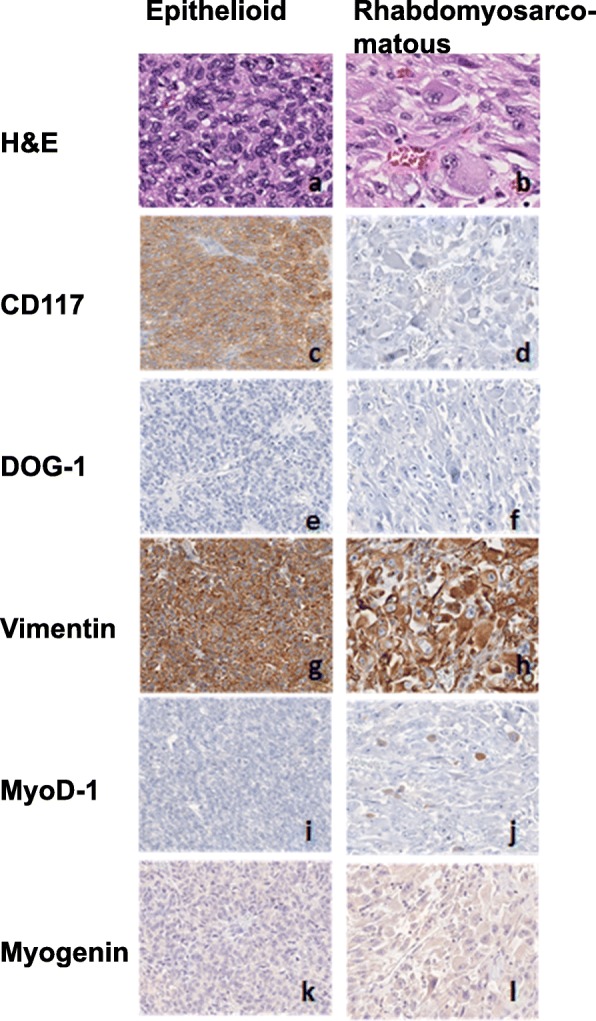
Morphologic and Immunophenotypic Features of Epithelioid and Rhabdomyosarcomatous Components of Tumor: left panel reveals H&E (400X) (**a**) and immunohistochemical stains of CD117, DOG-1, vimentin, MyoD1 and Myogenin- 200X (**c**, **e**, **g**, **i**, **k**) of the epithelioid cell components. Right panel reveals component H&E- 400X (**b**) and immunohistochemical stains of CD117, DOG-1, vimentin, MyoD1 and Myogenin (200X) (**d**, **f**, **h**, **j**, **l**) of the rhabdomyosarcomatous components

**Fig. 3:**
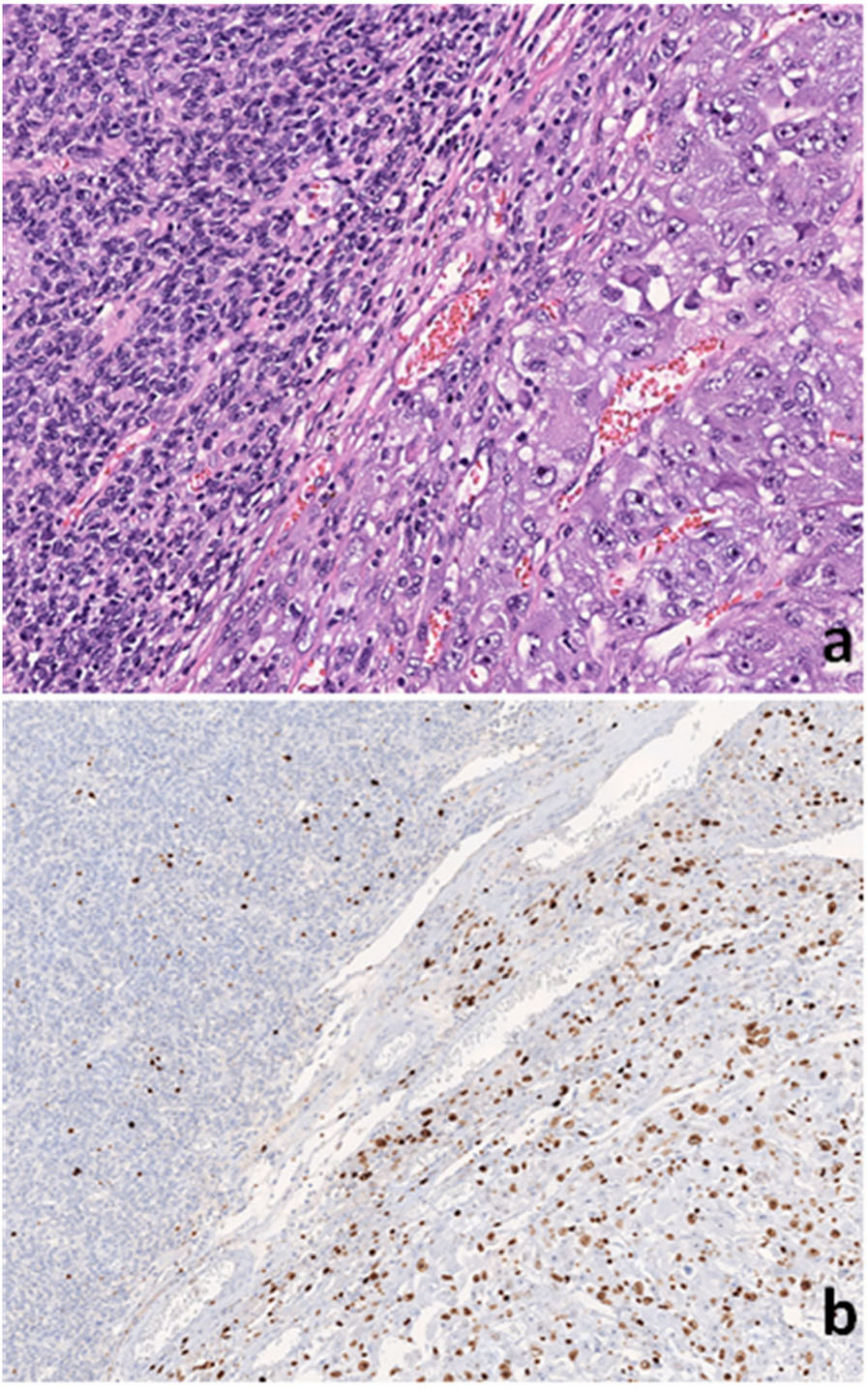
H&E Stain Depicting Abrupt Transition of Epithelioid and Dedifferentiated (rhabdymyosarcomatous) Components of GIST: epithelioid component (Left) and dedifferentiated (rhabdomyosarcomatous) (right) components of GIST at 100X magnification (**a**). Mitotic activity at abrupt transition area shown by immunohistochemical stain for proliferative index Ki-67 at 50X magnification (**b**)

Microscopic examination of the surgically resected tumor demonstrated extensive necrosis and hemorrhage. The viable tumor showed hypercellularity and an invasive growth pattern with spindle and epithelioid cells arranged in lobules. The majority of tumor cells were uniform with abundant eosinophilic cytoplasm, rare pleomorphic nuclear features, and occasional para-nuclear vacuoles (Fig. 2a). The epithelioid component of the tumor was positive for CD117 (Fig. 2c) and vimentin (Fig. 2g) but negative for DOG-1 (Fig. 2e), MyoD-1 (Fig. 2l), myogenin (Fig. 2k), desmin (not shown), and SMA (not shown) by immunohistochemical (IHC) stains. Interestingly, a small sarcomatoid component of the tumor (approximately 5–10%), abruptly adjacent to the classic GIST, showed rhabdomyosarcomatous differentiation, which revealed sheets of pleomorphic, large and bizarre rhabdoblasts with abundant eosinophilic material in the cytoplasm, irregular nuclei, prominent nucleoli, and increased mitotic activity (Figs. 2b, and 3). These sarcomatoid cells were diffusely and strongly positive for vimentin (Fig. 2h) and MyoD-1 (focally, cytoplasmic and nuclear stains, Fig. 2j), but negative for CD117 (Fig. 2d), DOG-1 (Fig. 2f), myogenin (Fig. 2l), desmin (not shown), and SMA (not shown) by IHC stains. Notably, there was a loss of CD117 expression in the dedifferentiated component of the tumor.

Additionally, sarcomatoid tumor cells had higher mitotic activity by immunostain for proliferative index Ki-67 compared to adjacent classic GIST tumor cells (Fig. 3a, b). The diagnosis of transformation of GIST with rhabdomyosarcomatous dedifferentiation was made. The tumor was a locally advanced high-risk GIST with risk of progressive disease based on tumor size, mitotic figures and the organ of origin (stomach). Only small areas of fibrotic change were present within the tumor, likely indicating minimal therapeutic response to IM. Mitotic rate was high (38 per 50 high power fields) in the transformed sarcomatoid component (Fig. 3b). The resection margins were all negative. Twenty lymph nodes were negative for tumor metastasis. The pathologic stage of this tumor was stage IIIB (pT4, PN0). Efficacy of treatment was difficult to evaluate due to the nature of the lesion showing hyper- and hypo-cellularity, myxoid stroma, fibrosis, and necrosis which is often seen in untreated GISTs as well. 90–95% of the tumor specimen was viable.

### Next-generation sequencing (NGS) analysis

The two different tumor types were identified on an H&E slide and the different tumor types were manually macrodissected from unstained slides. DNA was isolated using the QIAamp DSP DNA isolation kit (Qiagen, Inc). The DNA was converted into a sequencing library using the Illumina TruSight Tumor-26 (TST26) gene library construction kit and sequenced using the MiSeq sequencing system (Illumina), following the manufacturer’s instructions. The sequence was compiled and aligned using the MiSeq Reporter v2.6.2 and the variant call file was read and annotated using VariantStudio v.3.0 (Illumina).

Genes (exons) analyzed include the following [Gene (exon)]: AKT1 (2), ALK (23), APC (15), BRAF (11,15), CDH1 (8, 9, 12), CTNNB1 (2), EGFR (18–21), ERBB2 (20), FBXW7 (7–10,11), FGFR2 (6), FOXL2 (1), GNAQ (4–6), GNAS (6,8), KIT (9,11,13,17,18), KRAS (2–5), MAP 2 K1(2), MET (1,4,13-18,20), MSH6 (5), NRAS (2–5), PDGFRA (11,13,17), PIK3CA (1,2,7,9,20), PTEN (1–7,9), SMAD4 (8,11), SRC (10), STK11 (1,4,6,8), TP53 (2–5,7,8,10,11). Analytical sensitivity was 5%. Human Genome Version (Human hg19) was used for molecular data analysis.

Next-generation sequencing of the endoscopic biopsy specimen from an outside hospital showed an in-frame deletion of 6 nucleotides in exon 11 of KIT, with a variant allele frequency of 51%. This finding represents the classic component of GIST (prior to patient receiving IM treatment).

The surgically resected tissue was evaluated in order to investigate molecular differences between the classic and rhabdomyosarcomatous dedifferentiated components of the GIST. Adjacent tissue from abrupt transition areas (Fig. 3a, right) were macrodissected and subjected to NGS using the TST26 gene panel (Illumina). The variants identified are shown in Table 2.

**Table 2:** Variants identified in Classic and Rhabdomyosarcomatous (Rhabdo) GIST components

Gene	Nucleotide Change	Amino Acid Change	Consequence	VAF Classic GIST	VAF Rhabdo GIST
KIT	NM_000222.2:c.1670_1675delGGAAGG (6 nt)	Trp557_Val559delinsPhe	Inframe deletion	91.1	50.7
TP53	NM_000546.5:c.215C > G	Pro72Arg	missense	43	54.7
PDGFRA	NM_006206.4:c.2472C > T	none	synonymous	4.6 (ND)	25.4
KIT	NM_000222.2:c.2586G > C	none	synonymous	4.2 (ND)	26.0
PDGFRA	NM_006206.4:c.1701A > G	none	synonymous	99.8	99.8
APC	NM_000038.5:c.4479G > A	none	synonymous	51.2	49.9
EGFR	NM_005228.3:c.2361G > A	none	synonymous	93.8	70.8
MET	NM_001127500.1:c.1131C > T	none	synonymous	99.6	99.5

Gene, nucleotide change, amino acid change, consequence and variant allele frequencies (VAF) are shown. *ND* Not detected. The limit of detection for this assay is 5%, so VAFs below this threshold are not presented

Similar to the endoscopic biopsy specimen, NGS of the surgical specimen showed a KIT gene exon 11 in-frame deletion (a kinase activating variant) at relatively high levels in both the classic and dedifferentiated components. This variant is a 6-nucleotide exon 11 in-frame deletion (NM_000222.2:c.1670_1675delGGAAGG: p.W557_V559delinsF) of the KIT gene. This in-frame deletion removes amino acids 558 and 559 and changes the amino acid at position 557 from a tryptophan to a phenylalanine. Comparison of the sequence variants in the classic GIST vs. dedifferentiated GIST revealed no significant differences in nonsynonymous DNA variants. Four synonymous variants in PDGRFA, APC, EGFR, and MET, as well as a missense variant in TP53 were detected at similar levels in both GIST subtypes. No TKI resistant mutations were detected in exon 13, 17, or 18 of KIT or PDGFRA genes.

However, the dedifferentiated component of our specimen harbored two unique synonymous (silent) sequence variants when compared to the classic component. The unique synonymous variants are in the KIT (variant allele frequency, 25.99% vs. 4.21%) and PDGFRA (variant allele frequency, 25.42% vs. 4.57%) genes (Table 1). This indicates that there are at least two unique populations of cells present within this tumor. No variants were detected in the EGFR, NRAS, KRAS and BRAF genes; all of which have been previously reported to occur in dedifferentiated GISTs [2, 14, 15, 17].

### Cytogenetic analysis

Karyotype analysis was submitted to the cytogenetics and molecular lab at St Christopher Children’s Hospital (Philadelphia, PA).

Cytogenetic testing showed a complex male karyotype 55~84 < 3n>,XY,+X,-1,+ 2,+ 3,-7,+ 8,+ 8,+ 9,+ 11,-12,+ 12,-13,-14,-15,+ 15,-16,-17,-18,-19,-19,-20,+ 20,+ 21. Previous studies have also showed complex karyotypes in dedifferentiated rhabdomyoblastic components of GIST [14]. Previous studies have also shown the complex karyotype: 54–55, XY, +Y, + 1, del (1)(p21), + 6, + 7, + 7, + 12, _14, add (16), (p13), + 20, + 21, + 21[cp7]/46, XY [2].

## Discussion and conclusion

Approximately 80–85% of GISTs possess activating mutations of KIT, while 10% have activating mutations of PDGFRA. KIT and PDGFRA mutations are mutually exclusive [18]. GIST with a KIT (exon 11) mutation is more susceptible to IM therapy. Acquired mutations in KIT exons 13, 14, and 17 as well as PDGFRA exon 18 have been reported with long-term IM exposure [12]. Activating mutations in the BRAF (V600E) gene have been identified in 7% of KIT/PDGFRA wild-type GISTs located in the small intestine [19].

Studies have shown the morphologic and immunophenotypic changes with IM treatment of GISTs. “Resistant nodules” within response areas have been described on imaging in patients with treatment failure [14]. Furthermore, morphologically, spindle shaped tumor cells become epithelioid with variable CD117 expression [15]. Although less common, anaplastic or rhabdomyosarcomatous differentiation has also been seen with prolonged IM therapy [2, 14, 15, 17, 20, 21].

The dramatic morphologic and immunophenotypic changes seen in the reported seven cases with rhabdomyosarcoma involved abrupt transformation from classic CD117-positive tumor cells to CD117-negative tumor cells with marked anaplasia/pleomorphism [2, 14, 15, 17] (Table 1). Interestingly, of the seven cases reported, all but one showed KIT exon 11 mutations/deletions in both classic and dedifferentiated components of GIST suggesting the common origin of these two components [2, 14, 15, 17]. In one case, an exon 18 PDGFRA mutation was present [2]. In six of the seven cases, no additional mutations were identified in the IM resistant/dedifferentiated tumor areas including KIT, PDGFRA, KRAS, and BRAF [2, 14, 15, 17]. One case showed gain of KIT exon 13 mutation; however, this unusual differentiation did not show specific molecular changes [2]. This raises the possibility of activation of KIT-independent novel pathway contributing to loss of CD117.

Rhabdomyosarcomatous dedifferentiation of GIST has been reported in metastatic lesions of the omentum, ovary, peritoneum, mesentery and liver in patients with rapidly progressive IM-resistant metastatic disease. In all previously reported cases (7 cases), this sarcomatous dedifferentiation occurred after therapy with TKI with or without surgical debulking exclusively in the setting of metastatic disease. To our knowledge, we are presenting the first case of rhabdomyosarcomatous dedifferentiation in the absence of metastatic disease.

Interestingly, all reported cases of rhabdomyosarcomatous dedifferentiation (7 cases), as well as our case, reveal complete loss of CD117 immunoreactivity and preservation of the primary KIT gene exon 11 mutation or deletion [2, 14, 15]. Broadly, the mechanism of dedifferentiation in tumors (occurring de novo) may involve secondary genetic changes contributing to tumor progression and transformation, accompanied by morphologic and immunophenotypic changes. In advanced GISTs, morphologic and immunophenotypic changes do not always elucidate diagnosis, particularly when evaluating new lesions arising during treatment. However, molecular analysis of KIT mutations helps delineate the common origin of these lesions, providing a link between the primary tumor and recurrence/metastases [2, 14, 15].

Antonescu et al, presented eight cases of GIST with anaplastic dedifferentiation (non-rhabdomyomatous) and a CD117-negative phenotype. Five of the patients had no prior history of IM exposure, whereas three of the patients received long-term IM therapy. Treatment with IM can lead to altered morphology and loss of CD117 reactivity (as seen in three of the eight patients). Only in tumors of the three patients treated with IM did both classic and anaplastic components have a KIT mutation genotype [17]. Even though the same KIT mutation genotype was found in both classic and rhabdomyosarcomatous components of our patient’s GIST, de novo dedifferentiated GIST cannot be excluded since he received short-term IM treatment for only 3 months.

Seven cases published in the literature have shown tumor progression to anaplastic dedifferentiation after at least 8-month TKI treatment (400 mg QD or more doses) [2, 15, 17]. Similar to our case, Jiang’s case study also showed heterologous differentiation of the primary tumor (as opposed to metastases) following 8-month of TKI treatment [14]. These findings support the hypothesis that “resistant nodules” arise via clonal evolution [10]. Both our reported case and Jiang’s case demonstrate that tumor dedifferentiation following a short-term IM treatment course may be related to an alternative mechanism of TKI resistance. This molecular mechanism has not been established yet.

Molecular analysis of our patient’s GIST revealed equivalent genotypes in both the classic and dedifferentiated components, indicating a common origin rather than a collision tumor (an important differentiation in pathology practice) [15, 20]. Therefore, KIT mutational analysis is critical for accurate diagnosis of new lesions not expressing KIT or showing unforeseen immunohistochemical profiles and morphologic features.

As in our case, in all seven reported cases of rhabdomyosarcomatous transformation of GIST, the prevalence of KIT (exon 11) and PDGFRA activating mutations was preserved in each tumor’s dedifferentiated component. However, KIT protein expression (CD117) was either completely lost (7 cases) or significantly reduced (1 case) with transformation [2, 22]. Interestingly, all of these patients were maintained on TKI, with variable responses to therapy ranging from partial to complete (one patient). Some were continued on IM while others were transitioned to sunitinib for disease progression or lack of response to IM. When possible, surgical debulking was performed. In our patient with non-metastatic disease, complete surgical resection was performed. There were no TKI resistance mutations in the R0 resected tumor; therefore, IM therapy was initiated with planned maintenance for 3 years with periodic surveillance imaging.

Notably, in our patient, two synonymous (silent) variants were found in the dedifferentiated GIST. Thus, two unique populations of cells may be present within the sarcomatoid component. The low-level detection of these two variants in the classic GIST component likely reflects contamination of the classic GIST component with dedifferentiated GIST cells during macrodissection. Variants with frequencies below 5% are typically not reported as present in the specimen. These unique silent variants may reflect a higher DNA replication error rate and/or genetic instability in the dedifferentiated GIST. This is consistent with other studies that suggest that genetic instability, including loss of heterozygosity (LOH) and low level amplification of KIT contribute to CD117-negative GISTs [23]. Similarly, gene copy number abnormalities have been reported as the most common finding in dedifferentiated GISTs [17]. It is important to note that the TST26 library kit only analyzes a limited number of exons from 26 genes, so it is possible that there are other unidentified unique variants, synonymous and nonsynonymous, in dedifferentiated GIST. Overall, this data suggests that dedifferentiated GIST contains an increased number of DNA variants, as compared to classic GIST.

Although the abrupt transition from CD117-positive epithelioid to CD117-negative rhabdomyosarcomatous dedifferentiation is striking, the underlying molecular mechanism remains uncertain. The KIT activating variant is common in tumor cells in both the rhabdomyosarcomatous and classic GIST regions of the tumor, which strongly indicates that both components originated from the same primary tumor cell. Therefore, the loss of KIT expression is likely due to additional genetic or epigenetic changes that arose during rhabdomyosarcomatous progression.

In addition to gross chromosomal rearrangements, potential alterations could include mutations and/or small insertions/deletions that disrupt the open reading frame of the KIT gene or the splicing and processing of the KIT mRNA. Additionally, mutations within the KIT promoter may prevent transcription of the gene, preventing KIT protein expression. These theoretical DNA variants would be beyond the coverage of the NGS used in this study and thus not detected. Conversely, epigenetic changes, such as promoter methylation, could be responsible for the loss of kit expression in the rhabdomyosarcomatous tumor by blocking transcription of the KIT gene. In addition to the TP53 missense mutation, there were 4 synonymous variants (APC, MET, EGFR, and PDGFRA) that were present in both GIST phenotypes (Table 1) at similar variant allele frequencies. These likely represent germline variants, but this can only be confirmed by testing normal tissue from the patient. Secondary BRAF and/or KRAS mutations have been found coexisting with KIT or PDGFRA mutations in naive GIST, but their significance in inducing GIST dedifferentiation is unclear. No BRAF and KRAS mutations were seen in our patient [17]. Additionally, there were no mutations of KIT exons 13, 14, and 17 as well as PDGFRA exon 18, which have been reported after long-term IM treatment [12].

This study adds to the growing consensus that rhabdomyosarcomatous GIST progresses from a common GIST primary tumor. However, the role of IM treatment in this progression is uncertain and the short duration of IM treatment in this study supports the hypothesis that rhabdomyosarcomatous GIST progression is not a consequence of IM therapy.

In summary, our study shows morphologic and immunophenotypic rhabdomyosarcomatous dedifferentiation of a treatment refractory GIST without metastasis after short-term IM therapy. Together with reported cases, awareness of this rare clinical entity and its potential occurrence following TKI treatment could prevent a diagnostic pitfall. Molecular analysis provides valuable information exploring tumor origin and tumor progression and may assist with optimal treatment strategies in the future.

## Data Availability

Data sharing is not applicable to this article as no datasets were generated or analyzed during the current study.

## Abbreviations

GI: Gastrointestinal
GIST: Gastrointestinal stromal tumor
H&E: Hematoxylin and eosin
Human hg19: Human Genome Version
IM: Imatinib mesylate
LOH: Loss of heterozygosity
MYF-4: Myoenin
NCCN: National Comprehensive Cancer Network
NGS: Next gen sequencing
PDGFRA: Platelet-derived growth factor receptor alpha
RTK: Receptor tyrosine kinase
SMA: Smooth muscle actin
TKIs: Tyrosine kinase inhibitors
TST26: Illumina TruSight Tumor-26
